# Changes in Post-migration Living Difficulties Predict Treatment Outcome in Traumatized Refugees

**DOI:** 10.3389/fpsyt.2018.00476

**Published:** 2018-10-09

**Authors:** Matthis Schick, Naser Morina, Panagiota Mistridis, Ulrich Schnyder, Richard A. Bryant, Angela Nickerson

**Affiliations:** ^1^Department of Consultation Psychiatry and Psychosomatic Medicine, University Hospital Zurich, Zurich, Switzerland; ^2^Faculty of Medicine, University of Zurich, Zurich, Switzerland; ^3^School of Psychology, University of New South Wales, Sydney, NSW, Australia

**Keywords:** refugees, asylum seekers, refugee mental health, posttraumatic stress disorder, psychosocial interventions, post-migration living difficulties, social integration

## Abstract

**Background:** Refugee mental health is affected by traumatic stressors as well as post-migration living difficulties (PMLD). However, their interaction and causal pathways are unclear, and so far, no distinct treatment recommendations regarding exile-related stressors exist.

**Methods:** In a 3-year follow-up study, PMLD and symptoms of post-traumatic stress (PTS), depression and anxiety were examined in a clinical sample of severely traumatized refugees and asylum seekers (*N* = 71).

**Results:** In regression analysis, reduction in PMLD predicted changes over time in depression/anxiety, but not in PTS. The opposite models with PMLD changes as outcome variable proved not significant for PTS, and significant, though less predictive, for depression/anxiety.

**Conclusions:** In addition to well-established trauma-focused interventions for the treatment of PTS, psychosocial interventions focusing on PMLD might contribute to a favorable treatment response in traumatized refugees, particularly with regard to depression and anxiety.

## Introduction

There are currently over 65 million people displaced worldwide due to conflict, violence, and persecution (1). Refugees, asylum-seekers and other forcibly displaced populations typically report exposure to a high number of potentially traumatic events in their countries of origin and during displacement. These experiences are often prolonged, repeated, and interpersonal in nature and have a pervasive negative impact on mental health (2, 3). Accordingly, refugees have consistently been observed to present with high prevalence rates of trauma related mental health problems, particularly posttraumatic stress disorder (PTSD), depression, and anxiety (4–6).

In addition to past trauma, refugee mental health is also affected by difficulties arising *after* successful entry in a formally safe host country. Refugees usually face numerous daily challenges related to the post-migration environment, including those relating to lack of resources, family separation, social isolation, acculturation and discrimination, socioeconomic factors, and immigration and refugee policies. These displacement-related stressors have been shown to negatively impact on mental health over and above the effects of traumatic experiences (5, 7–9). Moreover, mental health problems of refugees are not limited to affected individuals, but can have a devastating impact on their families (10, 11).

While mental health problems due to traumatic and exile-related stressors can have a substantial impact on the psychological wellbeing of refugees and their close ones, they also lead to functional impairment. Refugees are usually expected, or legally obliged, to rapidly participate in the host society, particularly regarding language proficiency and financial independence. The process of social integration, however, implies high functional requirements in terms of cognitive and interpersonal capabilities, which refugees with psychological impairments are often not able to meet. Preliminary evidence shows that psychological impairment in refugees is associated with high levels of post-migration living difficulties (PMLD), and with poor social and economic integration (12–14). Prompt and appropriate treatment of mental health problems in refugees is therefore not only an ethical, but also an economic concern in hosting societies in the sense that inadequate or unavailable treatment can lead to substantial long-term social costs. Accordingly, a recent study of severely traumatized refugees with severe trauma related disorders found treatment to be economically beneficial on family income level after 3 years (15).

While recent research suggests that mental health problems of refugees and asylum seekers are best captured by models integrating pre- and post-migration factors (16–18), their complex interaction and causal pathways remain largely unclear. Accordingly, the therapeutic field is spread between two opposing assumptions: exponents of trauma-focused interventions consider trauma exposure the critical causal factor. They argue that an improvement of PTSD symptoms will lead to an improvement in functionality and better adaption to impending challenges of resettlement and acculturation. In contrast, for advocates of multimodal interventions, the attribution of causality focuses primarily on the overall stressful social and material conditions, suggesting a range of psychosocial interventions for the purpose of general stabilization, which, in turn, would allow better management of traumatic stress symptoms (8, 17). Evidence to date points to trauma-focused interventions being the most efficacious in reducing PTSD symptoms amongst refugees. In contrast, there has been little rigorous research investigating other approaches such as psychosocial interventions, and other diagnostic groups such as depression and anxiety disorders (17, 19).

Given the absence of a systematic framework to guide informed treatment decisions, research is urgently needed to examine the temporal and causal relationship between exposure to traumatic events and post-migration stressors, mental health, and real-life outcomes such as education, employment, or social integration (20). In view of the growing public health dimension of mental disorders in refugees, predictors and treatment moderators should be investigated in order to identify individuals for which specific approaches are indicated or those who fail to benefit from best practice interventions. Understanding the direction of causality between changes in psychological symptoms and changes in post-migration stressors would directly inform services tasked with supporting refugees in their host environment.

This study investigated the association between change in PTSD, depression and anxiety symptoms and change in post-migration stressors in a sample of refugees receiving treatment at a torture survivors' outpatient clinic. Participants were assessed at baseline and after a 3-year follow-up. To our knowledge, this is the first longitudinal study in a clinical sample of severely traumatized refugees to examine the relationship between PMLD and treatment trajectories. We hypothesized that participants would demonstrate reduced PTSD, depression and anxiety symptoms between baseline and follow-up assessment. We secondly hypothesized that improvements in PMLD scores between baseline and follow-up would relate to greater decreases in PTSD, depression and anxiety at follow-up. Finally, due to the exploratory character of the study and in consideration of a circular model, we had no hypothesis regarding the direction of causality between PMLD and psychiatric symptoms.

## Methods

### Participants

The sample consisted of participants of an earlier, cross-sectional study (*N* = 134, for details s. 12) who were re-assessed after a projected follow-up time of 3 years. Participants were refugees and asylum-seekers from a variety of countries of origin. They were in treatment in two psychiatric outpatient units for victims of torture and war in Zurich or Bern, Switzerland. Treatment included a variety of manualized trauma-specific as well as non-manualized unspecific psychotherapeutic interventions and medication, depending on symptom profiles and subjective focus of distress, individually adapted to the patients' needs and capacity with regard to content and dosage. In addition to treatment, all participants were offered social counseling, which individually addressed respective PMLD including accommodation, legal, financial or language problems, among others. Patients aged 18 years or older and speaking one of the study languages (German, English, Turkish, Arabic, Farsi, or Tamil) were included in the study. Current psychotic symptoms, severe dissociative symptoms, and acute suicidality led to exclusion. At follow-up, *N* = 44 participants could not be contacted any more, and N = 19 refused to participate. Only completers of the follow-up assessment were included in this study, resulting in a sample size of *N* = 71. Data collection took place between 2012 and 2013 (T1), and between 2015 and 2016 (T2), respectively. The follow-up time was *M* = 39.6 (*SD* = 4.6) months.

### Measures

All measures used in the study were translated and back-translated, if necessary, by accredited translators in accordance with gold-standard translation practices (21). Discrepancies were rectified jointly by the research team and independent bilingual individuals who were experienced in working with health-related questionnaires.

Exposure to traumatic events was indexed using a measure derived from combining the trauma event lists of the Harvard Trauma Questionnaire [HTQ, (22)] and the Posttraumatic Diagnostic Scale [PDS, (23, 24)]. Overall trauma exposure was represented by a count of the number of traumatic event types (ranging from 0 to 23) experienced by each participant.

Symptoms of PTSD in the past month were measured using the PDS. The PDS contains 17 items assessing the occurrence of DSM-IV symptoms of PTSD in the previous four weeks on a 4-point Likert-scale (range 0–3). To account for the anticipated changes of the diagnostic criteria of PTSD in DSM-5 (25), which was not yet published at the beginning of the data collection, four additional items were added and one item was excluded, as it was no longer included in the DSM-5 diagnostic criteria. A comparison of DSM-IV and DSM-5 diagnostic criteria using part of this sample has been published elsewhere (26). We computed a total continuous score of PTSD symptoms (Range 0–60) and employed a DSM-5 based diagnostic algorithm to determine likely PTSD caseness. The scale has been used with numerous refugee groups [e.g., (27, 28)]. Cronbach's alpha at T1 in this study was 0.94.

Depression and anxiety in the past week were measured using the Hopkins Symptom Checklist HSCL-25 (29). This 25-item scale measuring psychological distress has been employed with a number of refugee groups [e.g., (30, 31)]. We computed subscale scores for anxiety and depression as well as a total continuous score (range 1–4). The validity of the often-used 1.75 cut-off criterion for clinically relevant anxiety and depression has been evaluated in relation to different populations around the world and found to be accurate (29, 32). Cronbach's alpha at T1 was 0.94.

Living difficulties were measured with the Post-Migration Living Difficulties Checklist (33, 34) adapted to the Swiss context (12). This 17-item scale (range = 0–68) examined the extent to which post-migration challenges had been a problem for the individual over the past 12 months. Items are rated on a five-point scale (0 = not a problem to 4 = very serious problem). Items scored at least 2 (“moderately serious problem”) were considered positive responses, yielding a total count of living difficulties. The PMLD scale has consistently been identified as a predictor of mental health among displaced populations (12, 31, 35).

### Procedure

The study was approved by the Ethics Committees of the Cantons of Zurich and Bern. Written informed consent was obtained, with participants being informed they were free to withdraw from the study without influence on future treatment. Questionnaires were applied using a therapist-assisted computer-based assessment tool MAPSS [Multi-Adaptive Psychological Screening Software, (36)]. In MAPSS, self-report questionnaires are presented on an electronic tablet in both written and auditory form in the respondent's mother tongue. Assessments were supervised by a clinical psychologist or a masters-level student of clinical psychology. Participants were reimbursed CHF 40 (approx. USD 40) for participation.

### Data analysis

All statistical analyses were conducted using SPSS Version 25. Descriptive statistics are given in terms of means and standard deviations in continuous variables, and counts and percentages for categorical variables. Changes over time were calculated for all variables. Separate linear regression analyses (method: enter) were conducted for PTSD severity, depression/anxiety, and PMLD as outcome variables, respectively. Variables entered into the models included gender, trauma exposure, visa status (secure vs. insecure) and employment status (employed vs. unemployed) at T1, and change in PMLD between T1 and T2.

## Results

### Sample characteristics

Sample characteristics are described in Table 1. Participants were from a variety of countries of origin, including Turkey (*N* = 42, 59.2%, with *N* = 34, 47.9% being Kurdish), Iran (*N* = 6, 8.5%), Sri Lanka (*N* = 6, 8.5%), Iraq (*N* = 4, 5.6%), Bosnia (*N* = 3, 4.2%), and other countries (*N* = 10, 14.1%, e.g. Afghanistan, Somalia, etc.). The education level was rather high with 75% of the sample having at least a high school degree. Despite an average stay in Switzerland of 10 years at baseline, only *N* = 14 (19.7%) had a fulltime or part-time employment, and roughly one fourth still had an insecure visa status. Participants reported severe lifetime trauma exposure with an average of 12.5 (*SD* = 4.3) potentially traumatic event types (PTE) experienced. The most commonly reported PTE included torture (85.9%), enforced isolation from others, combat situations (78.9% each), and imprisonment (77.9%). The least commonly reported were sexual assault by a stranger (26.8%) and sexual contact when younger than 18 (14.1%).

**Table 1:** Sample characteristics (*N* = 71).

**Variable**	**T1**	**T2**
	***M* (*SD*)/*N* (%)**	***M* (*SD*)/*N* (%)**
Age	44.55 (9.05)	47.96 (9.08)
Gender (male)	61 (85.9)	
Length of time in Switzerland	10.41 (6.70)	13.87 (7.11)
**MARITAL STATUS:**		
Single	14 (19.7)	12 (16.9)
In a relationship/married	49 (69.0)	45 (63.4)
Widowed/divorced	8 (11.3)	14 (19.7)
**EDUCATION:**		
Primary school not completed	7 (9.9)	7 (9.9)
Primary school completed	(15.5)	(15.5)
High school	29 (40.8)	29 (40.8)
Bachelor's degree or college	19 (26.8)	19 (26.8)
Postgraduate degree	5 (7.0)	5 (7.0)
EMPLOYMENT^*^:		
Full-time	8 (11.3)	9 (12.7)
Part-time	6 (8.5)	15 (21.1)
Unemployed	42 (59.2)	27 (38.0)
Retired/homemaker	14 (19.7)	20 (28.2)
**VISA STATUS:**		
Asylum seeker	11 (15.5)	1 (1.4)
Temporary visa	6 (8.5)	5 (7.0)
Permanent visa	16 (22.5)	18 (25.4)
Residency	28 (39.4)	32 (45.1)
Citizenship	10 (14.1)	15 (21.1)
PTE exposure (HTQ)	12.5 (4.3)	

*PTE, potentially traumatic event types*.

* *Employment N = 70*.

A comparison on key variables between participants, who completed both time points versus those who completed Time 1 only, indicated significant differences exclusively regarding age and length of time in Switzerland, but not regarding symptoms scores, trauma exposure, and PMLD. Specifically, those who completed Time 2 were significantly older (*t* = 3.08, *p* = 0.002) and had lived in Switzerland for a longer time (*t* = 2.65, *p* = 0.01).

### Symptom scores

Symptom scores at baseline and follow-up are shown in Table 2. Symptoms of PTSD and depression/anxiety were significantly lower at follow-up. While 93% of the participants reported clinically relevant depressive symptoms at T1, only 62% did so at T2. With regard to anxiety, 88.7% were above the cut-off at T1, and 53.5% were above cut-off at T2. At T1, 47.9% had a probable PTSD diagnosis according to DSM-5 criteria as compared to 45.1% at T2. Fourteen participants (19.7%) reported symptom levels compatible with a new probable diagnosis of PTSD.

**Table 2:** Symptom severity (M/SD) of PTSD, depression and anxiety at T1 and T2.

	**T1**	**T2**	***t (df)***	***p***
PTSD (PDS total score)	34.52 (13.63)	29.62 (12.21)	2.99 (70)	0.005
Depression (HSCL depression subscale)	2.73 (0.59)	2.1 (0.63)	7.02 (70)	<0.001
Anxiety (HSCL anxiety subscale)	2.71 (0.70)	1.86 (0.50)	12.36 (70)	<0.001
HSCL_total	67.76 (14.87)	49.94 (11.67)	9.86 (70)	<0.001

*PTSD, Post-traumatic stress disorder; HSCL-25, Hopkins Symptom Checklist. P–values are not controlled for multiple comparisons*.

### Post-migration living difficulties

Frequencies of PMLD types at baseline and follow-up are shown in Table 3. More than half of PMLD types improved significantly along the treatment trajectory. Among the PMLD types with the most substantial improvement were issues related to visa status (“not being recognized as a refugee,” “being fearful of being sent back to your country of origin in the future,” “difficulties in interviews with immigration officials”). Frequently occurring PMLD types without improvement were mostly related to family members left back home (“worries about family back home,” “being unable to return to your home country in an emergency,” “separation from family”).

**Table 3:** Post-migration Living Difficulties experienced as moderately serious, serious or very serious (*N* = 71).

**PMLD type**	***n*** **(%)**		***P*^d^**
	**T1**	**T2**	
Mean	10.03 (4.3)	7.79 (3.63)	<0.001^e^
Loneliness, boredom or isolation	61 (85.9)	52 (73.2)	0.022
Worries about family back home	61 (85.9)	58 (81.7)	0.453
Being unable to return to your home country in an emergency^a^	53 (75.7)	52 (73.2)	1.000
Difficulty learning German	54 (76.1)	43 (60.6)	0.013
Separation from family	54 (76.1)	50 (70.4)	0.289
Difficulties with employment^b^	45 (65.2)	34 (47.9)	0.017
Communication difficulties	47 (66.2)	36 (50.7)	0.007
Being fearful of being sent back to your country of origin in the future	46 (64.8)	14 (19.7)	<0.001
Difficulties obtaining financial assistance^a^	39 (54.9)	31 (43.7)	0.012
Difficulties obtaining appropriate accommodation	40 (56.3)	33 (46.5)	0.143
Not enough money to buy food, pay the rent or buy necessary clothes^b^	39 (54.9)	37 (52.1)	0.804
Discrimination^a^	36 (50.7)	30 (42.3)	0.146
Worries about not getting access to treatment for health problems	38 (53.5)	29 (40.8)	0.022
Difficulties in interviews with immigration officials	27 (38.0)	14 (19.7)	0.007
Not being recognized as a refugee	28 (39.4)	6 (8.5)	<0.001
Conflicts with social workers/other authorities	25 (35.2)	21 (29.6)	0.344
Ethnic conflicts^c^	19 (26.8)	13 (18.3)	0.109

a *n = 70*.

b *n = 69*.

c *n = 68*.

d *McNemar's test*.

e *t-test*.

### Predicting change in symptom levels over time

The results of the regression analysis for PTSD and depression/anxiety controlled for gender, trauma exposure, visa status, education and changes over time in PMLD are shown in Table 4. Reduction in PMLD predicted changes over time in depression/anxiety as assessed with HSCL-25, accounting for 17.7% of the variance. The overall model was not associated with changes in PTSD, though the individual predictor was significant.

**Table 4:** Summary of the multiple regression analyses (*N* = 71).

**Outcome variable**	**Independent variable**	***B***	***SEB***	**β**	***T***	***P***	***F***	***p***	***R*^2^**	**Adjusted *R*^2^**
PTSD score change							1.52	1.00	0.11	0.037
	Gender	1.63	4.96	0.04	0.33	0.743				
	Employment	−1.47	4.26	−0.04	−0.34	0.732				
	Trauma exp.	0.51	0.40	0.15	1.25	0.214				
	Visa status	−2.38	3.95	0.07	−0.60	0.549				
	PMLD change	1.49	0.64	0.28	2.32	0.023				
HSCL-25 score change							3.97	0.003	0.237	0.177
	Gender	7.93	4.89	0.18	1.62	0.110				
	Employment	3.72	4.21	0.09	0.88	0.381				
	Trauma exp.	0.79	0.39	0.22	1.98	0.052				
	Visa status	−0.80	3.89	−0.02	−0.20	0.838				
	PMLD change	2.38	0.63	0.41	3.76	<0.001				

*HSCL-25, Hopkins Symptom Checklist; PTSD, Post-traumatic stress disorder; PMLD, Post-migration living difficulties*.

In order to examine directionality of findings, we analyzed the opposite models with PMLD changes as outcome variable, and change in PTSD and depression/anxiety as the predictor variables, which proved not to be significant [*F*_(5, 64)_ = 1.300, *p* = 0.275] for PTSD, but significant for depression/anxiety [*F*_(5, 64)_ = 3.086, *p* = 0.015], respectively, explaining 13.1% of the variance.

## Discussion

This 3-year longitudinal study investigated the association between changes in symptoms of PTSD, depression/anxiety and changes in post-migration living difficulties (PMLD) in a sample of severely traumatized refugees receiving treatment and social counseling in a specialized outpatient center.

Our hypothesis of generally lower symptom scores of PTSD and depression/anxiety at follow-up was fully supported by our findings. Along with mental health improvements, we found a significant reduction in more than half of the examined PMLD types as well as of the mean PMLD scores. Remarkably, while PTSD symptom scores on average improved under treatment, 14 participants (19.7%) who scored below the cut-off of probable PTSD diagnosis at baseline, exceeded this cut-off at follow-up. As all participants had a clinically established diagnosis of PTSD at the beginning of treatment (being a requirement for treatment uptake), it seems that after initially benefitting from therapy until the baseline assessment, some patients later experienced increased posttraumatic stress again. This finding has been described in other studies investigating refugees in the post-migration (37, 38). It can be hypothesized that, in our sample, additional traumatic experiences occurring during follow-up, and/or actualization of past traumatic experiences, e.g., due to political aggravation in the countries of origin, as was the case in Turkey, accounted for elevated PTS scores. Further longitudinal research is required to investigate this hypothesis.

The relationship between the observed improvements in PMLD and improvements in symptom scores was examined in various regression models with changes in PMLD, symptoms of depression/anxiety (as measured by the HSCL-25), and PTSD, respectively, as outcome variables. The best predictive model was that with depression/anxiety as outcome und PMLD changes as independent variable (17.7% of variance explained, *p* ≤ 0.001), while the models with changes in PTSD and PMLD as either outcome or independent variable proofed not significant. These findings connect to earlier studies showing that, in general, trauma-related factors seem to explain more variance in rates of PTSD, while post-migration or displacement-related stressors appear to particularly influence rates of mood and anxiety disorders, over and above the effects of past trauma [for overview s. (7, 8)]. In addition to the existing cross-sectional evidence, our longitudinal data suggest that, in a treatment-seeking sample of severely traumatized refugees and asylum seekers in the post-migration, improvements in PMLD predict a favorable treatment trajectory with regard to depression and anxiety, and could therefore be valuable targets of therapeutic interventions. This finding, though preliminary in nature and in need of replication, contributes to several fields of discussion and potentially has substantial implications:

A first implication appears with regard to explanatory models and causality. There is solid evidence that refugee mental health is related to both pre-migration, traumatic as well as post-migration stressors (7, 17). The scientific discourse on how these aspects are causally related, however, is prototypically divided between two opposing models (8, 39, 40): The war-exposure model on the one hand focuses on traumatic experiences and consecutive PTSD. Post-migration living difficulties are at least partly considered a consequence of trauma-related psychological impairment and supposed to be manageable after symptoms have improved. Accordingly, trauma-focused interventions within a cognitive-behavioral framework are considered first-line approaches. On the other hand, representatives of multimodal or psychosocial interventions primarily target general sources of distress, particularly exile-related stressors, with the objective of secondary psychological stabilization.

While the direction of causality is clear regarding traumatic experiences and trauma-related disorders, it is much less clear with PMLD and psychiatric symptoms: post-migration stressors could promote mental disorders such as depression and anxiety, and the latter could lead to functional impairment and, therefore, to PMLD. The findings in our sample now suggest that PMLD have a permissive or even causal role regarding the development of depression and anxiety in traumatized refugees, rather than vice versa. From a clinical perspective, this makes sense as many of the most distressing PMLD, such as insecure visa status, separation from family members, or restrictive asylum policies, are unswayable for affected persons, even if psychological impairment is successfully treated. However, while changes in PMLD were predictive for changes in depression/anxiety, the inverse model was statistically significant as well, though less predictive. The assumption of a unidirectional model seems therefore less conclusive than a circular model with PMLD contributing to mental health problems, and, to a minor degree, vice versa.

A second implication touches the question of how refugee mental health should best be addressed in the context of service provision. While the effectiveness of trauma-focused interventions with regard to symptoms of PTSD is well documented, this applies only to a minor degree to other psychiatric disorders such as depression and anxiety disorders, and (so far) not to psychosocial interventions (17). Our findings provide preliminary support that addressing PMLD via psychosocial interventions within a multimodal framework, including trauma-focused approaches, could enhance treatment response with regard to depression and anxiety and, therefore, justify the delivery of respective treatment options. Remarkably, in our sample, not all PMLD types were equally amenable to change. Most significant improvements were found in stressors related to visa status. Unfortunately, this issue is entirely out of reach for therapeutic as well as psychosocial interventions. On the other hand, no significant improvement at all could be achieved with regard to stressors related to separation from family members in participants' home countries. Therefore, the most promising targets of psychosocial interventions seem to be those post-migration stressors related to social integration such as language and employment.

A third important implication relates to immigration policies and social integration. In many high-income countries, the barriers for asylum seekers are high in order to avoid pull-factors. Many aspects of daily life of asylum seekers such as long asylum procedures, placement in camps, restrictive access to labor market, or prohibition of family reunion are intentionally harshened by the authorities. In contrast, after obtaining a residency permit, refugees are usually expected to rapidly engage in the host societies, particularly regarding language proficiency and financial independence. An earlier, cross-sectional study on partly the same sample found a high correlation between psychological impairment and integration difficulties (12). In addition, our longitudinal findings suggest that host societies could facilitate successful social integration of mentally ill asylum seekers and refugees by complementing timely and appropriate treatment with psychosocial interventions targeting PMLD. This is in line with a recent study of severely traumatized refugees which found multimodal treatment including social counseling to foster economic integration on family income level (15). Conversely, restrictive policies aiming at establishing stressful living conditions for refugees and asylum seekers might fuel PMLD and consecutive mental health problems, with negative consequences for social integration.

This study has a number of limitations. First, the sample size was small. This may have reduced our statistical power in uncovering relationships between variables. Second, participants were examined in different stages of therapy, rather than in proper pre-post-assessments, which may limit the comparability of trajectories. Due to the clinical character of the sample, our findings are not generalizable to subclinical refugee populations. Third, though we used transculturally validated measures whenever available, participants were from numerous cultural backgrounds, and thus, it was not possible to use measures validated with each cultural group. Yet, the use of a tablet-based therapist-assisted assessment tool allowed participants to be assessed in their respective mother tongues independently of their level of education. Finally, self-report measures were implemented instead of clinician-administered diagnostic interviews and an objective index of PMLD. It may be the case that those participants with high symptom scores were more likely to perceive objective stressors as more stressful.

## Conclusions

This study provides preliminary evidence for the causal role of PMLD with regard to mental health problems of refugees and asylum seekers. Our findings support an ecological model of refugee mental health, which suggests that both pre-migration and post-migration stressors contribute to mental health outcomes (16). In addition to well-established trauma-focused interventions for the treatment of PTSD, our data suggest that psychosocial interventions focusing on PMLD might be able to contribute to a favorable treatment response, particularly with regard to depression and anxiety, and may therefore be a legitimate and valuable add-on in a multimodal treatment approach. Future research should examine (a) what PMLD topics are causing the most distress and impairment, (b) what topics are amenable to therapeutic interventions, and (c) what interventions are the most effective ones in order to achieve the greatest relief. Policy makers should recognize the role of daily stressors in contributing to psychological distress and their negative impact on social integration. It may be in the interest of host societies to support aid agencies, caseworkers or settlement service providers in addition to psychological treatment.

In addition, psychosocial interventions may be of particular interest in view of the fact that the highest share of displaced persons in need of support are not located in high-income countries, but in conflict, post-conflict or neighboring countries without access to (appropriate mental) health services. Non-medical approaches such as accessing social support and problem management, provided by helpers without health-professional background, are urgently needed (41). The required task-shifting from highly-qualified specialists to less specialized workers with fewer qualifications may be more likely to succeed with interventions addressing daily stressors than specific diagnoses (42).

## Author contributions

MS was involved in the conception of the study, in the interpretation of the data and the drafting of the manuscript. NM was involved in the conception and design of the study, the acquisition, analysis and interpretation of the data, and the drafting of the manuscript. PM was involved in the analysis and interpretation of the data, and the drafting and revision of the manuscript. US, RB, and AN designed the study and contributed to the manuscript. All authors read and approved the final manuscript.

### Conflict of interest statement

The authors declare that the research was conducted in the absence of any commercial or financial relationships that could be construed as a potential conflict of interest.
